# Supplementary material for: Multilevel logistic regression modelling to quantify variation in malaria prevalence in Ethiopia
Source: PLoS One. 2022 Sep 29;17(9):e0273147. doi: 10.1371/journal.pone.0273147 (PMC9521912; doi:10.1371/journal.pone.0273147)
Supplement: S1 File — (PDF) [file pone.0273147.s001.pdf]

Supplementary Materials for "Multilevel Logistic Regression Modelling to Quantify Variation in Malaria Prevalence in Ethiopia"

Bereket Tessema Zewude[1,◑‡*], Legesse Kassa Debusho[1◑], Tadele Akeba Diriba[1] ,

1Department of Statistics, University of South Africa c/o Christian de Wet Road & Pioneer Avenue, Private Bag X6, Florida 1710, Johannesburg, South Africa

◑These authors contributed equally to this work.
*Corresponding Author: bereket.tessema2010@gmail.com
‡Current Address: Department of Statistics, College of Natural and computational Science, Wolaita Sodo University, Wolaita Sodo, Ethiopia

Supplementary Materials

This supplementary provides additional details and results in table form for the multilevel logistic regression modelling to quantify variation in malaria prevalence in Ethiopia presented in the main manuscript.

Model selection

To compare the models fitted for the individual / household characteristics, we computed the likelihood ratio statistic, the Akaike information criterion (AIC) and the Bayes information criteria (BIC). Furthermore, we have assessed the significance of fixed effects or covariates using Type III tests. We first fitted the model with ten covariates selected by stepwise variable selection technique. In this model we have considered only the variables with VIF less or equal to 5. The results of this model are presented in Table A.

Table A: Type III tests for the Covariates or predictor variables for the binary GLMMs.

Variables	Degrees of freedom	χ^2	p-value
Age	1	5.81	0.0159
Gender	1	5.55	0.0185
Household size	1	0.68	0.4113
Number of months since sprayed	1	2.72	0.0993
Household uses mosquito nets	1	5.53	0.0187
Availability of windows	1	2.14	0.1438
Type of toilet facility	2	3.75	0.1533
Main material of house roof	2	0.62	0.7350
Main source of drinking water	2	4.01	0.1346
Main material of house wall	2	1.46	0.4825
Fit statistics			
-2 Log Likelihood	1658.29		
AIC (smaller is better)	1690.29		
BIC (smaller is better)	1751.32		

The next step following the above inferences on the coefficients of the fitted model is to remove covariates which are implausible. However, it is recommended to do this by removing one covariate at a time. The new, reduced model should be compared to the old or larger model using the likelihood ratio test. Note that the reduced model is nested in the largest model. The list of variables removed at each subsequent step together with the p-value of the type III test, the statistics -2 Log Likelihood, AIC and BIC after removing the variable are displayed in Table B.

Table B: Results of applying backward variable elimination: variables removed, p-value of the type III test, -2 Log Likelihood, AIC and BIC for the binary GLMMs.

Step	Variable removed	p-value	-2 Log Likelihood	AIC	BIC
1	Main material of house wall	0.5553	1659.05	1687.05	1740.45
2	Household size	0.4134	1660.54	1684.54	1730.31
3	Type of toilet facility	0.1367	1661.00	1683.00	1724.96
4	Main source of drinking water	0.1769	1666.79	1684.79	1719.12
5	Type of toilet facility	0.2453	1667.24	1685.24	1719.57

Results of logistic regression models

Table C: Estimated regression coefficients (SE), p-value of Wald χ^2 and fit statistics for the logistic regression model.

Independent variables	Degrees of freedom	Fitted models: Model M_1 Estimate (SE)	Model M_1 p-value	Model M_2 Estimate (SE)	Model M_2 p-value
Child related variables					
Intercept	1	-3.879 (0.115)	< 0.0001	-0.556 (0.399)	0.1641
Age	1	-0.015 (0.005)	0.0032	-0.013 (0.005)	0.0088
Gender, Female	1	-0.155 (0.070)	0.0270	-0.164 (0.071)	0.0209
Windows exist, Yes	1	0.244 (0.072)	0.0007	0.349 (0.076)	< 0.0001
Number of months since sprayed	1	0.136 (0.022)	< 0.0001	0.068 (0.025)	0.0072
Mosquito nets, Yes	1	0.447 (0.079)	< 0.0001	0.227 (0.084)	0.0073
Drinking water (Ref Unprotected)					
Protected source	1	-0.331 (0.139)	0.0175	-0.398 (0.142)	0.0049
Piped water	1	0.145 (0.112)	0.1978	0.132 (0.113)	0.2430
Toilet facility (Ref No facility)					
Flush toilet	1	0.459 (0.106)	< 0.0001	0.352 (0.112)	0.0017
Pit latrine	1	-0.092 (0.095)	0.3285	-0.092 (0.099)	0.3531
Region (Ref Amhara)					
Oromiya	1			-0.786 (0.115)	< 0.0001
SNNP	1			0.520 (0.102)	< 0.0001
Median Altitude	1			-0.002 (0.0002)	< 0.0001
Fit statistics					
-2 log(Lik)		1903.20		1786.60	
AIC (smaller is better)		1923.20		1812.60	
BIC (smaller is better)		1994.55		1905.35	
