# Supplementary material for: Multilevel logistic regression modelling to quantify variation in malaria prevalence in Ethiopia
Source: PLoS One. 2022 Sep 29;17(9):e0273147. doi: 10.1371/journal.pone.0273147 (PMC9521912; doi:10.1371/journal.pone.0273147)
Supplement: Attachment — Submitted filename: Authors Response to Reviewers Comments - PONE-D-20-29896.pdf [file pone.0273147.s002.pdf]

Response to the of referees' comments on the manuscript:

"Multilevel logistic regression modelling to quantify variation in malaria prevalence in Ethiopia" by Bereket Tessema Zewude, Legesse Kassa Debusho; Tadele Akeba Diriba

Manuscript ID: PONE-D-20-29896

Dear Editor,

The first version of the manuscript was submitted to PLOS ONE in September 2020, and we have received the response from the Academic Editor with reviewers' comments within few weeks. We are very grateful to the Academic Editor for the summary version of reviewers' comments and encouraging words that motivated us to resubmit the revised version as a new submission. We are also very grateful to the reviewers for the thorough review of our manuscript and for the insightful and detailed comments. We hope we have addressed all the points that the Academic Editor and referees have made and provide the following summary of our changes in the first version in case if it is required.

Academic Editor

Dear Dr. Zewude,
… Major concerns were related to methodological flaws that may have affected the conclusion. As strongly recommended by Reviewer #1, the authors must exclude highly correlated covariates before regression analysis; otherwise, those variables will bring the same information to the model. Also, it seems critical to exclude non-significant variables and reduce dimensionality of models. The conclusion should be based on the best-selected model. In addition, a significant amount of issues should be adjusted and/or clarified in the MS. A copy of the reviewers' comments was included for your information.

Although the MS is not currently publishable, we believe that the paper has merit and can be considerate in the future after substantial revision. Thus, if the authors decide to submit the manuscript again it should be as a new submission. It means that a new editor and/or new reviewers may handle the resubmission. Despite of that, resubmissions are linked to previous submissions, so original comments may be considered.
I am sorry that we cannot be more positive on this occasion, but hope that you appreciate the reasons for this decision.

Yours sincerely,
Luzia Helena Carvalho, Ph.D.
Academic Editor
PLOS ONE

Authors' response:

In the revised version of the manuscript all the comments raised by the two reviewers have been addressed and incorporated. The revised manuscript has been modified based on the comments and responses for the comments were prepared separately for each reviewer in this report. Specifically, in the revised version of the manuscript we have excluded highly correlated covariates before fitting the multilevel logistic regression models and those which are statistically nonsignificant, and the discussion and conclusion are based on the best-selected model.

Reviewer 1 Comments

1. Based on data from 3 regional states of Ethiopia, the authors used multilevel logistic models with random effects to assess factors that affect malaria RDT outcomes. Statistical modelling of malaria surveillance data can be explored to characterize drivers of transmission heterogeneity and provide evidence for the rational deployment of control interventions. However, the current version of the manuscript has major issues as depicted below.

   Before the regression analysis, authors should verify if covariates are not correlated. If two covariates are highly correlated, they bring the same information to the model. Regarding models (M1 and M2) presented at Tables 2 and 3, they include many not significantly variables (e.g., windows, number of sleeping rooms and household wealth index). These variables can affect the overall inference and conclusions. There are many statistical procedures which can exclude non-significant variables and reduce dimensionality of models. I strongly recommend the authors to use different variable selection methods in order to select the best model and, after it, draw the conclusions based on the best selected model. In order to select the best model, the authors should also consider some statistical measure (e.g., AIC, BIC) and confirm if results are satisfactory with some model diagnostics.

   Authors' response:

   We have considered the reviewer recommendation and revised the model fitting process and variables selection using statistical measures, namely likelihood ratio test, AIC and BIC. This is explained in the Results section under the heading "Fitted multilevel models" and provided supporting results, please see Lines 251-275 on page 9.

2. The authors added interaction just between two covariates (household wealth index and household toilet facility). What was the justification for it? Results presented at Figure 1 do not show any clear tendency.

Authors' response:

Due to the revised model selection process, since the household wealth index is highly correlated with other variables and its effect was statistically nonsignificant it was excluded from the fitted models. Therefore, the interaction effect was not considered in the revised version of the manuscript.

3. I do not understand why the authors presented the information from Table 1. How does it contribute to the analysis?

   Authors' response:

   Table 1 is excluded from the revised version.

4. It is not clear how VPC and MOR formulas were derived. Is there a reference for it? Why was the 75th percentile of the normal distribution considered in the MOR formula?

   Authors' response:

   References for the theoretical or mathematical derivation of VPC and MOR formulas are provided in the revised version, please see Lines 235-236 (page 8) and Line 241 (page 9).

5. In the subsection "Population-average regression coefficients and odds ratios", the point about the shrinking factor is not clear and completely vague. Please, improve the formulation, add it to the Methods Section and reformulate the text.

   Authors' response:

   We revised the statement related to the shrinkage factor and moved the topic to the Methods Section, please see Lines 198-204 on page 7.

Reviewer 2 (Julius Nyerere Odhiambo) Comments

1. Abstract
   The objectives as specified in the abstract seems to be at variance with the title of the manuscript. The malaria indicator survey data needs to be specified i.e. by year and geographical extents.

   Authors' response:

   We have made corrections in the Background part of the abstract to address the two issues.

2. Introduction
   Needs to be rewritten coherently. The authors need to start with a global perspective, Africa and then narrow down to Ethiopia. Many thoughts spanning from vector control (Line 1-2) to current statistics (Line 6-8) could be separated and highlighted in independent paragraphs.
   Many loose statements are prevalent in the introduction which needs to be substantiated.
   Line 7: “However, the rate of reduction of malaria 6 mortality was slower in the period 2016–2018 than in the period 2010–2015…” This need to be referenced.
   What do the authors mean by conventional binary logistic regression model? (Line 34 – 35). I suggest the authors highlight that in the context/nature of the data collected it might not be the robust modelling approach to employ. How about comparing both models?
   The main objective of the paper needs to be rewritten to capture the need for an optimal model in assessing the factors (Line 66 – 72)

   Authors’ response:

   We have made necessary changes in the Introduction section to address the above comments, please see Lines 1-27 and Lines 41-46 on pages 2 and 3.

   Further, we have compared the standard logistic regression with the model that we have recommended in this study in the Results section, please see Lines 264-275 on page 9.

   The objectives paragraph is rewritten in the revised version to address the issue raised by the Second reviewer on Lines 73-79 on page 3.

3. Materials and methods
   This section should be simplified and trimmed to the methods applied directly to the study. Other details should be discussed in the result section (See lines 197 – 211)
   How were the explanatory variables (individual level and cluster level) used in the model selected?
   The sampling weights used need to be justified (Line 182 – 189).

   Authors’ response:

   We have discussed the methods that we have used in the study in the Statistical methods subsection under the Material and Methods section, please see Lines 157-245 on pages 5-9.

   The explanatory variables selection process was discussed in the Results section (please see Lines 251-272 on page 9) and the comment on the sampling weights is discussed in the Sampling weights subsection under the Material and Methods section (please see Lines 183-191 on pages 5-6).

4. Results

Am surprised to see model formulations in this section. (Lines 255 – 256, lines 351 - 352).
The null hypothesis in (Lines 258 – 260) should be expressed in a way that suits a general audience.
Could the significant variables mentioned (line 292) be stated outrightly?

Authors' response:

These comments also shaped our revised presentation. The model formulations moved to the Statistical methods subsection in the revised version, please see Lines 191-197 on pages 6-7.

We have rephrased the test on the variance components, please see Line 276 on page 9.

The significant variables in the study are listed in the Results section, please see Lines 258-261 on page 9.

5. Discussion
Has this method been applied in other malaria endemic zones in SSA? If so, is there a variation with the results presented here?
Perhaps the implication of the study to policy would be important if outlined.

Authors' response:

The authors could not find literature that applied the methods used in the current study on malaria endemic zones in SSA. However, we have compared our findings related to effects of various explanatory variables with published results in the Discussion section.

The implications of the current study to police outlined as recommendations, please see Lines 465-472 on page 15.
