# Supplementary material for: Multilevel logistic regression modelling to quantify variation in malaria prevalence in Ethiopia
Source: PLoS One. 2022 Sep 29;17(9):e0273147. doi: 10.1371/journal.pone.0273147 (PMC9521912; doi:10.1371/journal.pone.0273147)
Supplement: Attachment — Submitted filename: Response to Reviewers.pdf [file pone.0273147.s003.pdf]

Response to the Academic Editor and Reviewer # 1 comments on the manuscript:

“Multilevel logistic regression modelling to quantify variation in malaria prevalence in Ethiopia” by Bereket Tessema Zewude, Legesse Kassa Debusho; Tadele Akeba Diriba

Manuscript ID: PONE-D-21-12914

Dear Editor,

We are very grateful to the Academic Editor and Reviewer # 1 for the thorough review of our manuscript and for their insightful and detailed comments. The marked-up copy of our manuscript that highlights changes made to the original version has been uploaded as a separate file labeled “Revised Manuscript with Track Changes”. We hope we have addressed all the points that the Academic Editor and Reviewer # 1 have made and provide the following summary of our changes.

Academic Editor Comments

1) Overall the English language is good but the manuscript needs a final copy-edit before publication. E.g. 2.4 million (not millions) in the abstract. Line 36 should read ‘may have *more* similar’. There are other small errors.

Authors’ response:

In the revised version of the manuscript, this and other errors that were identified by the Academic Editor and Reviewer # 1 as well as by the authors have been corrected.

2) Introduction: Please update the malaria burden numbers from 2018 to more recent data (e.g. using the latest World Malaria Report)

Authors’ response:

We have considered the Academic Editor recommendation and revised the malaria burden numbers from 2018 to 2019 using the World malaria report 2020, please see Lines 9-15 and 23-25 on page 2.

3) Table 1. Please briefly explain in the legend the difference between the 3 models. The table should be broadly understandable to the reader without referring to the main text.

Authors’ response:

We have made a change in the legend to address the Academic Editor suggestion, that is, the following statement was used as a legend:

"Estimated regression coefficients (SE) and variance components for the models with cluster-specific random effects b_j only (M_0), with the individual / household-level predictors and b_j (M_1), and M_1 with cluster-specific predictors (M_2)."

4) The abstract is rather hard to follow for a generalist reader. Please consider rewriting this sentence: "The main objective of this study was to quantify the variance component of cluster effects and cluster characteristics on individual malaria rapid diagnostic tests (RDT) outcome." Perhaps you could say something like 'to quantify the extent of clustering of malaria infection as assessed by RDT'.

   Similarly the results section of the abstract is hard to read. Can you name some of the most important variables for explaining the clustering, which you found?

Authors' response:

We have considered the Academic Editor recommendation and revised the Abstract. The changes are marked by red on page 1 on the revised version of the manuscript.

5) I suggest moving the final paragraph which discusses malaria climate further up in the discussion, since this is a key point. Please reference other studies which have sought to explain malaria prevalence by both climate variables as well as MIS variables. For example the Malaria Atlas Project is a major group which has been working on this problem for many year. E.g. see the map tab on the Malaria Atlas Project forecasts of malaria prevalence for Ethiopia https://malariaatlas.org/trends/country/ETH What are the differences in the variables used for analysis in this study or other similar geospatial models compared to yours?

Authors' response:

These comments also incorporated in the revised version. The final paragraph in the "Conclusion" section of the old version was moved to the last part of the "Discussion" section, please see Lines 457-511 on pages 14-15. In addition, based on the Academic Editor's comment, we have added some information related to the Malaria Atlas Project (MAP), the limitations of our work, and what MAP provides. Then made recommendation for further research to address the gaps.

6) I suggest removing the phrase ‘best alternative’ for analysis on line 398, since there are many other sophisticated methods which seek to do this, e.g. geospatial modelling as referenced above.

Authors’ response:

The phrase “best alternative” has been removed in the revised version, please see Line 406 on page 13.

Reviewer # 1 Comments

1) Minor points

   My main problem with the manuscript in it's current state is that the policy importance of the main aim of the analysis is not well explained and is not trivially obvious.
   What policy implications does a large (or small) between-cluster variance component have?
   I can imagine some.
   Maybe it suggests that we are still missing covariates important for predicting malaria.
   If so, what might those covariates be and what are the barriers to collecting that data?
   Is it that nonlinear models and interaction terms are needed?
   Or is it that there are cluster level drivers that we will never be able to measure?
   What does this mean for policy, that we an never make excellent predictions of malaria or that the only way to guide resource distribution is to measure malaria prevalence in ever cluster in a country?

Authors’ response:

We have incorporated the following in Lines 474-492 on page 15 of the revised version:

“As discussed in the result section, the results of this study show that there is still high variation between survey enumeration areas (SEAs) or clusters in malaria RDT outcomes after taking into account the effects of individual / household characteristics and the two cluster effects. The variation could be due to bio-ecological or human factors or other covariates that are important for predicting malaria prevalence but not included in the study. These factors may help to guide malaria control interventions and improve their efficiency and effectiveness, and hence may lead to more effective public health strategies and could have important policy implications for health promotion.”

We hope this and the information that followed address the Reviewer # 1 concern.

2) Relatedly, while you cite some references that use random effects models, you don't cite any estimates of between-cluster variance from any other study. While I recognise that these estimates might be difficult to find in many papers, if this is the main aim of the analysis, any previous attempts at estimating this value should be mentioned, if nothing else to help us set our expectations. Have previous estimates found that between-cluster variation is very high or very low?

Authors' response:

We have added the following literature review information in the "Introduction" section in Lines 57-67 on page 3 to address the issues raised by Reviewer # 1:

"There are two multilevel studies in Ethiopia, Peterson et al. [15] included village as a cluster and concluded that village-level effects were important and Woyessa et al. [16] considered household as a cluster and investigated individuals and household effects but their study was conducted within one city in Oromiya region. The studies in Peru [17] and Cambodia [18] also included household as a cluster in their multilevel analyses. The later showed the importance of household-level risk factors in explaining differences in infection prevalence between households and between villages. Whereas the former compared risk factors in community-specific models and reported malaria prevalence differences between communities were influenced by individual and household variables but variances of the random household-level effects were too small."

3) I'm not sure if this point is relevant but in some senses, the cross-validation error in models of malaria prevalence are a measure of the same quantity. It is the amount of malaria prevalence that cannot be predicted with the covariates in the model.

Authors' response:

The point has merit, but we think that it is not related to the objectives of the current study.

4) The paragraph in lines 80-84 seems very unecessary to me. The structure of the paper is totally standard.

Authors' response:

We have taken out the above lines in the revised version.

5) Lines 243-245. I'm not sure what this line means. Are you saying that you needed to seperately calculate the weights and tell PROC GLIMMIX to use them? Or that you have provided the weights in an attached dataset? Or something else.

Authors' response:

We have rephrased the statement to make it clearer in revised version, please see Lines 255-257 on page 9.

6) The section in lines 251-257 that explain how variable selection was performed should be in the methods somewhere.

Authors' response:

We have moved the variable selection information to "Methodology" section in the revised version, please see Lines 204-209 on page 7.

7) Lines 367-395. Given that this section is the main result (according to the aims of the analysis stated in the abstract and intro) I think it should go near the beginning of the results section. Unless I have misunderstood, the hypothesis tests in line 276 are part of the same result (is the variance component zero, if not how big is the variance component). So bringing these two sections together would improve the structure.

Authors' response:

We have considered the Reviewer # 1 recommendation and we have moved the "Quantify variation: Variance partition coefficient and Median odds ratio" section to Lines 335-364 on page 12. However, to keep the consistency of the results presentation, we have not moved the hypothesis tests for variance components, or we have not joined the two sections. The first subsection of "Results" section focuses on fitted multilevel models where we have discussed the tests for model parameters including the variance components.

8) In the abstract in the results (word limit allowing), I think it would be useful to state the result that 55% of the variability in malaria outcomes could be attributed to the differences between SEAs. This to me is the most useful statement of the results.

Authors' response:

We have considered the Reviewer # 1 recommendation and have amended the abstract accordingly, please see the "Results" section of the abstract.

9) Line 418: I am glad that you spend some time to discuss the surprising result that mosquito net use predicts increased malaria prevalence. However I think you have perhaps missed a likely cause of this estimate. I would guess that the positive relationship is because nets are delivered to areas with high malaria burden and people are more likely to use the nets if they feel malaria is a big risk to them. This argument does however raise the problem that the causality might be reversed here (malaria causes nets).

Authors' response:

We have found the phrase in red in the above Reviewer # 1 recommendation very relevant and we have included it in the revised version, please see Lines 426-427 on page 14.

10) Line 197: You have written the sigma2b is an estimate of what *could* be explained. Quite subject but I would think of it as what *needed to be* explained. The random effect could explain everything if you removed everything.

Authors' response:

We have made a correction in Line 203 on page 7 as it has been suggested by Reviewer # 1.

11) Language issues

We have also made all the language related corrections as suggested by Reviewer # 1. Below we listed the line number and page where the suggested corrections have been made.

- Abstract, methods. A sample of 9272 individuals were *used* in this

  The correction has been made in Line 1 of "Methods" section of the abstract on page 1.

- Abstract, conclusions. The methods used in this study have allowed *us* to make

  The correction has been made in Line 1 of "Conclusions" section of the abstract on page 2.

- Line 28: the survey design organizes the.

  The correction has been made in Line 28 on page 2.

- Line 29: *They* then select. Or something like that.

  The correction has been made in Line 29 on page 2.

- Line 36: or enumeration area, may have more similar.

  The correction has been made in Line 36 on page 2.

- Line 44: This model also does not allow us to study the association

  The correction has been made in Line 44 on page 3.

- Line 46: The intra-cluster correlation can *give* useful. Or something.

  The correction has been made in Lines 46-47 on page 3.

- Line 54: regression models allow *analysts* to take into

  The correction has been made in Line 54 on page 3.

- Line 59: within-cluster.

  The correction has been made in Line 70 on page 3.

- Throughout, check whether within-cluster and between-cluster have hyphens.

  We have made changes wherever necessary.

- Line 65: as a set of control variables, *the analysis* may provide information *on* how

  The corrections have been made in Lines 76 and 77 on page 3.

- Line 70: Also, it does not allow *analysts* to examine *the* influence of

  The corrections have been made in Line 81 on page 3.

- Equation 2 and 3: I think the squared term in the denominators should come after the i|j. {w_{i|j}}^2 if you use latex.

  The corrections have been made in the two equations as it was suggested on page 6.

- Line 186: The *results from the* weighted analyses across

  The correction has been made in Line 192 on page 6.

- Line 187: and variance *parameters* of the

  The correction has been made in Line 193 on page 6.

- Line 188: results obtained *were* slightly

  The correction has been made in Line 194 on page 6.

- Line without a number after equation 5: identical for all the SEAs and bj *quantifies*

  The correction has been made in the second line from the bottom on page 6.

- Line without a number before equation 6: The third model *was* defined

  The correction has been made in the third line after the equation on page 7.

- Just after line 233: The VPC measures *the* proportion

  The correction has been made in the last line of page 8.

- Line 315: to *have* had malaria than

  The correction has been made in Line 321 on page 10.

- Line 332: their interpretations *are* natural

  The correction has been made in Line 369 on page 13.

- Line 426: This *implies* that

  The correction has been made in Line 433 on page 14.

- Line 446: explanatory variables *were* introduced

  The correction has been made in Line 453 on page 14.

- Line 447: Hence, there is still a large

  The correction has been made in Line 454 on page 14.

- Line 456: have allowed *us* to make

  The correction has been made in Line 518 on page 16.

- Line 461: Such *an* approach yield*s*

  The corrections have been made in Line 523 on page 16.

- Line 483: environment *are* interrelated, interpretation

  The correction has been made in Line 473 on page 15.

- Ref 29. The first author is Rabe-Hesketh S I believe.

  The correction has been made in Reference 33 on page 18.

- Ref 35 Type in neighbourhood.

  The correction has been made in Reference 39 on page 18.
