# Supplementary material for: Multilevel logistic regression modelling to quantify variation in malaria prevalence in Ethiopia
Source: PLoS One. 2022 Sep 29;17(9):e0273147. doi: 10.1371/journal.pone.0273147 (PMC9521912; doi:10.1371/journal.pone.0273147)
Supplement: Attachment — Submitted filename: Response to Reviewers.pdf [file pone.0273147.s004.pdf]

Response to the Academic Editor and Reviewer # 1 comments on the manuscript:

"Multilevel logistic regression modelling to quantify variation in malaria prevalence in Ethiopia" by Bereket Tessema Zewude, Legesse Kassa Debusho; Tadele Akeba Diriba

Manuscript ID: PONE-D-21-12914R1

Dear Editor,

We are very grateful to the Academic Editor and Reviewer # 1 for the thorough review of our manuscript and for their insightful and detailed comments. The marked-up copy of our manuscript that highlights changes made to the original version has been uploaded as a separate file labeled "Revised Manuscript with Track Changes". We hope we have addressed all the points that the Academic Editor and Reviewer # 1 have made and provide the following summary of our changes.

Academic Editor Comments

I see things missing, starting with the rationale, the population of interest, who was tested for malaria, everybody in the household? A sample?

Authors' response:
In the revised version of the manuscript, we have included information that explains why the three regions were considered, please see Lines 166-169 on page 4 Further, the other part addressed in the "Response and explanatory variables" section, please see Lines 173-177 On page 4.

1) Rationale: The authors do not provide the rationale for studying MIS2011 when a more recent MIS2015, https://www.malariasurveys.org/documents/Ethiopia_MIS_2015.pdf, is available. They should discuss the rationale why this might be still interesting. They mention the survey as ref [6] but they do not say why they have not used MIS 2015. They should also explicitly mention the trends. They mention the trend from 2015-2019. What happened from 2011 to 2015? That should come first since this is precisely after this study.

Authors' response:

The authors have requested for 2015 malaria indicator survey data, the response that we got was "the archived data didn't appear to be full and in the required format". Then EPHI has shared few datasets, which has no information on the survey design, such as strata, cluster and sampling weights. Since this information vital for our research, we could not consider the MIS2015 data that was provided to us. The authors assume including this justification in the manuscript is not appropriate.

We have considered the Academic Editor recommendation and revised the "Introduction" section to address the trend issue, please see Lines 57-75 on page 2.

2) The literature revision does not include multilevel studies using MIS. They exist, should be mentioned and acknowledged and compared in the discussion. There is in particular a study using Ethiopia MIS 2015, https://doi.org/10.1186/s12889-020-09560-1 . See also https://doi.org/10.1016/j.ijid.2020.12.062 . Also the authors should not oversell the novelty of the study. Using multilevel modelling is standard in many fields and using sample weights with complex survey structure is a requirement in order to get results representative at the National level. It is not a plus to use them. It is just wrong if you do not use them here. It might be right if a different sampling strategy is used. Overall the introduction can be shortened considerably

Authors' response:

We have considered the Academic Editor recommendation and revised the "Introduction" section to include the above suggested literatures and others, please see Lines 115-119 on page 3.

However, please note that in the first article the authors have used the sampling weights only to compute the descriptive statistics not for modelling purpose, whereas in the second articles the authors fitted unweighted multivariate models. Otherwise, we fully agree that "*using sample weights with complex survey structure is a requirement in order to get results representative at the National level*". As we have mentioned in the "Introduction" section, however this is not the case in most of the existing literatures.

3) There is a need to edit the language. Please make a thorough language check. Example of sentences that can be improved: "The results also show that the individuals vulnerability to malaria infection decreases significantly with increase in age". Maybe "increasing age" or "for older, maybe separate sentences

Authors' response:

In the revised version of the manuscript, this and other errors that were identified by the authors have been corrected.

4) Your main result on cluster-level variance should refer to the best model. And that is the model with covariates. However the abstract states "About 55% of the variability in malaria RDT outcomes of individuals could be attributed to the difference between clusters. However, when accounting for the individual / household- and cluster-level characteristics, part of the variability which is relevant at the cluster-level became lower.". Please provide only the figure for the best overall model according to AIC or BIC (say explicitly which criteria you are

using) mentioning that the percentage variability is “after controlling for ….”. On the other hand, you might report M1 instead of M2 since M2 precisely aims at explaining what is behind the cluster variation (with no change in the individual level controls).

Authors’ response:

These comments also incorporated in the revised version. The Abstract was corrected, the results of the best model were presented in “Results” section (please see Lines 361-430 on pages 10-13) and the “Discussion” section also focused on these results (please see Lines 477-495 & 499-519 on pages 14-15). The fit statistics or model selection criteria are presented in Table 1 and since M2 is the best model its results are presented in Table 2 separately.

5) Make clear in the abstract the population you are analyzing: Eg: “A sample of 9272 individuals” [representative of the National population? The adult population? This is never very clear]. Also: why are you focusing only on three states? How are these states compared to the National average?

Authors’ response:

We have made changes in the “Methods” section of the abstract to address the above comment and similar changes were also made in the “Study data” section, please see Lines 24-30 on page 1.

6) The referee is concerned with the lack of policy feedback gained from the multilevel analysis. While it is true that more recent data being available, this has less of an impact, the policy consequence of finding that a large proportion of variance remains at the cluster level is that policy should focus on those hotspots of malaria. In order for the analysis to be useful what would be interesting is to know where these malaria hotspots are located. You should provide a map showing the cluster random effects to guide action and comment on them (do they move along regional lines, are they very local, …).

Authors’ response:

Based on the above comments we have added some results from spatial clustering analysis supported by a figure which contains hot-spots areas or districts. Please note that due to the geographical coordinates of sample households we could not able to produce a map of hotspots with regions or districts names in one plot, we don’t have a shape file used for the MIS rather we presented it as point plot, please see Figure 1 on page 13. Then we have made comments or recommendations by referring the hot-spots and significant factors,

7) In the introduction you mention two levels of effects, the cluster and the household. However, the methods section only has random effects at the cluster level. This should be listed as a limitation since there are obvious correlation among malaria status at the household level. Why are you not including this? That would strengthen the paper since part of the currently described as cluster variance must be household-level variance and you can potentially separate one from the other?

Authors' response:

In the revised section, we have given explanation in the "Multilevel logistic regression model with random effects" section why we did not include a random effect for a household-level, please see Lines 229-235 on page 5. It reads as follows:

"In addition, there is correlation among malaria status of individuals living in the same households. These introduce intra-class correlation, which is a measure of the degree of similarity among malaria status of members of the same cluster, i.e. SEA or household. However, in the dataset used in this study only one member per household was tested for malaria from 47.12% households in the sample. This could introduce large number of missing cases if a within household variation considered."

8) I have been unable to look at the supplementary material and it is fundamental to see whether PLOS ONE statistical guidelines are being followed since there are many missing elements in the main text. Please provide it in an edited format, eg. Pdf. Not tex.

Authors' response:

The authors apologize for uploading the text version of the Supplementary material in the previous submission, now we have provided in pdf format.

9) In table 1 provide fit statistics for model 0.

Authors' response:

In the revised version, we have provided the fit statistics for all fitted models in Table 1, please see page 10.

10) The names of the models are different in table 1 and 2. Is model 2 model 1 and model 3 model 2?

Authors' response:

We have made correction in the revised version, please see Tables 1 and 2 on pages 10 and 12, respectively.

Reviewer # 1 Comments

The authors have made a number of language edits in line with the editors comments and my own. These are all fine.

None of my comments in my original review were very important. However, the comment that I thought was most important related to setting the study into it's policy context. Some of the results, such as estimating which factors (age, temperature etc.) are related to high malaria, have obvious policy implications, but are also the same thing that people have been estimating for years. The big novelty in this study that I could see was the focus on estimating the between cluster variance after accounting for covariates (this is stated as such in line 456). ***However, the policy importance of the between cluster variance wasn't clear to me; how does a Ministry of Health use this information to guide malaria control for example?***

In response the authors have added "The variation could be due to bio-ecological or human factors or other covariates that are important for predicting malaria prevalence but not included in the study. These factors may help to guide malaria control interventions and improve their efficiency and effectiveness, and hence may lead to more effective public health strategies and could have important policy implications for health promotion."

This states that the results are important for policy but still don't say how or why they are important. Again, ***what specific actions might a ministry take that they wouldn't have had taken without this information?***

Authors' response:

The main purpose of estimating between cluster variance in the study was to assess between cluster variation and if there is a variation what percent of it explained by the covariates in the model. The above quoted response was to mention that the unexplained heterogeneity after considering the individual / household and SEA characteristics or covariates possibly due to those variables not included in the model. Now in the revised version, we have recommended specific action related to significant factors or covariates and results from spatial clustering analysis, specifically hotspots.
