# Supplementary material for: Multilevel logistic regression modelling to quantify variation in malaria prevalence in Ethiopia
Source: PLoS One. 2022 Sep 29;17(9):e0273147. doi: 10.1371/journal.pone.0273147 (PMC9521912; doi:10.1371/journal.pone.0273147)
Supplement: Attachment — Submitted filename: Response to Reviewers .pdf [file pone.0273147.s005.pdf]

Response to the Academic Editor comments on the manuscript: "Multilevel logistic regression modelling to quantify variation in malaria prevalence in Ethiopia" by Bereket Tessema Zewude, Legesse Kassa Debusho; Tadele Akeba Diriba

Manuscript ID: PONE-D-21-12914R2

Dear Editor,

We are very grateful to the Academic Editor for the thorough review of our revised manuscript and for the insightful and detailed comments. The marked-up copy of our manuscript that highlights changes made to the original version has been uploaded as a separate file labelled "Revised Manuscript with Track Changes". We hope we have addressed all the points that the Academic Editor has made and provide the following summary of our changes.

1) Line numbers are missing in the manuscript with track changes. This makes it difficult to edit the manuscript. Please include them next time.

Authors' response:

The authors apologize for uploading the revised manuscript without line numbers in the previous submission. The authors have included the line numbers in the current revised version of the manuscript.

2) Abstract: I noted the rationale was missing and it is still missing. The background section cannot start as it does with "The 2011 Ethiopian National Malaria Indicator Surveys (EMIS) data used in this study have a multilevel structure where individuals in selected households are nested within cluster and this may result in dependent data". You should start stating the purpose of your study and why is it relevant. The purpose also cannot be to apportion the variance by itself. It must be to apportion the variance of some measure of interest. You have reformulated the introduction but not the abstract.

Authors' response:

We have considered the Academic Editor recommendation and revised the "Background" section of the Abstract to include the above comments, see page 1.

3) You repeat twice “malaria rapid diagnostic test outcomes” in the background. Better use “prevalence of malaria”, or even in the first appearance “the prevalence of malaria

Authors’ response:
We have considered the above comment and made changes in the “Background” section of the Abstract, see page 1.

4) You declare twice in the abstract that you focus on three regions but you do not list them. Please do so. Note also the clumsy writing of the first mention on “Further, the study was also interested in assessing factors that affect the malaria rapid diagnostic test outcomes of household members in three major regions of Ethiopia.”. That cannot be “also” an interest. You are ONLY studying the prevalence of malaria in these 3 regions, not anywhere else. This is connected to the previous comment. The background section in the abstract has to be completely rewritten.

Authors’ response:
We have rewritten the “Background” section in the Abstract, see page 1.

5) Note in the introduction you are also stating (p.3) similar things to the abstract. Please change also the introduction stating clearly the specific rationale at the beginning as it was suggested in the previous revision.

Authors’ response:
Based on the above comment, we have restructured the “Introduction” section, see pages 2-3.

6) In the rest of the abstract you are not providing the concrete results or conclusions. Please state your MAIN results (maximum of 3 or 4) in a quantitative way.

Authors’ response:
Based on the above comments we have rewritten the “Results” and “Conclusion” sections, see page 1.

7) Typos still present Eg: Supplemantary material is called, several times, “Supplementery Material”.

Authors’ response:

Based on the above comments we have replaced the phrase “Supplementery Material” by “Supplementary Material” in the current revised version of the manuscript and in the Supplementary material file wherever necessary.

8) The supplementary material needs to be thoroughly revised. It cannot be so verbose. What you are describing is the application of a backwards elimination algorithm. You do not need to describe each step in detail with all the estimates. Usually backwards elimination is carried out directly through some algorithmic procedure and you just need to report the variables you sequentially remove and their effect on the selection criteria (eg: AIC, BIC). It seems you are doing it by hand based on the p-value of a joint significance test. You could report the first step (the complete model), a table with the list of variables removed at each subsequent step together with the p-value of the type III test and the AIC or BIC after removing the variable, and the last step.

Authors’ response:

The authors made changes in the current revised version of the manuscript “Supplementary Material” as it was suggested in the above comment.

9) There is a need to copy-edit the manuscript. One more instance: “These three regions were considered due to the data sharing police of Ethiopian Public Health Institute that since we are not part of EMIS project team we had a limited access to the survey data for our research.” I’d suggest “The choice of these three regions was driven by the data-sharing limitations imposed by the Ethiopian Public Health Institute, the owner of the data”.

Authors’ response:

In the current revised version of the manuscript, we have made changes in Lines 111-112 on page 3 as it was suggested above.

10) The description of the target population and the sampling strategy needs to go in the data section, not in the response and explanatory variables section.

Authors' response:

The description of the target population and the sampling strategy was moved to the "Study data" section Lines 114-118 on page 4 in the current revised version of the manuscript.

11) The policy implication in p.14 is unwarranted, "Therefore, the regional government should frequently provide IRS to households in malaria hot-spots areas and the population at risk, and further educate people about its advantage" given that you do not find a significant effect.

Authors' response:

Due to the Academic Editor Comment stated in Item 13, in the current revised version of the manuscript contains additional information to strengthen the police implication that the authors stated in the previous submission. Please see results in Table 1 on page 11 and Lines 459-464 on page 17.

12) You are not commenting in the discussion the apparent contradictory result that those with mosquito nets are more likely to be infected. Why should that be so? Is this in line with the literature and with results from Ethiopia?

Authors' response:

In the current revised version of the manuscript, the authors have made comment, please see Lines 470-481 on page 17. It reads as follows:

"The current study showed that there was a positive association between household has mosquito nets and the prevalence of malaria in the study regions, this contradicts results reported in malaria literature. However, this association was statistically nonsignificant. The positive associate of having mosquito nets with the prevalence of malaria could be because mosquito nets are delivered to areas with high malaria burden or due to either households did not treat the nets with insecticide or inappropriate use of the nets or perhaps an individual or a household member was exposed to mosquito bites

during other times of the day or evening when the net was not in use. This also could be due to household members did not get proper training on how to use the nets from local public health workers. In addition, less than half of the sampled households in the study regions had no mosquito nets, this may increase the vulnerability of individuals living in the households which had no mosquito nets to malaria infection."

13) The paper is missing a table one with the proportions and mean values of variables, possibly by region, possibly according to the value of the dependent variable. Its absence makes interpretation very hard. Eg: Are mosquito nets very prevalent and its absence is telling us something about local conditions? Is spraying common… Please include it and use to it to inform the interpretation of results and the discussion.

Authors' response:

The table was provided on page 11 in the current revised version of the manuscript and the results in this table were discussed in Lines 260-295 on pages 9-10.

14) Figure 1 has too small a type. It cannot be read.

Authors' response:

The size of the fonts for Fig 1 (b) legend were increased
